# LncRNAs in cardiac hypertrophy: From basic science to clinical application

**DOI:** 10.1111/jcmm.15819

**Published:** 2020-09-08

**Authors:** Lei Liu, Donghui Zhang, Yifei Li

**Affiliations:** ^1^ Key Laboratory of Birth Defects and Related Diseases of Women and Children of MOE Department of Pediatrics West China Second University Hospital Sichuan University Chengdu China; ^2^ State Key Laboratory of Biocatalysis and Enzyme Engineering School of Life Science Hubei University Wuhan China

**Keywords:** cardiac hypertrophy, cardiomyocytes remodelling, long non‐coding RNA, targeting therapy

## Abstract

Cardiac hypertrophy is a typical pathological phenotype of cardiomyopathy and a result from pathological remodelling of cardiomyocytes in humans. At present, emerging evidence demonstrated the roles of long non‐coding RNAs (lncRNAs) in regulating the pathophysiological process of cardiac hypertrophy. Herein, we would like to review the recent researches on this issue and try to analysis the potential therapeutic targets on lncRNA sites. Studies have revealed both genetic mutations related hypertrophic cardiomyopathy and the compensative cardiac hypertrophy due to pressure overload, inflammation, endocrine issues and other external stimulations, share a common molecular mechanism of ventricular hypertrophy. The emerging evidence identified the abnormal expression of lncRNAs would leading to the impairment the function of sarcomere, intracellular calcium handling and mitochondrial metabolisms. Several researches proved the therapeutic role of lncRNAs in preventing or reversing cardiac hypertrophy. With the development of delivery system for small pieces of oligonucleotide, clinicians could design gene therapy approaches to terminate the process of cardiac hypertrophy to provide better prognosis.

**Key points**

1. Several lncRNAs participate in the regulation of pathophysiological process in cardiac hypertrophy, interacting with the genes involving sarcomere, mitochondria and intracellular calcium transition.
2. Current evidence demonstrates the lncRNAs could be a promising target to attenuate the process of cardiac hypertrophy or even reverse the pathological phenotype. And a series of animal and few large animal experiments have been done to identified the potential activity of particular lncRNAs as a treatment alternation.
3. Non‐viral and viral strategies are the most common concepts to realize the gene therapy. A set of pre‐clinical experiments identified the capability of lncRNAs in treating cardiac hypertrophy. And this review illustrates a completed practical process to validate a lncRNA‐based gene therapy from basic science to clinical application.

## 1 INTRODUCTION

Cardiac hypertrophy is considered as a pathological phenotype of hypertrophic cardiomyopathy (HCM) or a result from cardiomyocytes’ remodelling due to a series of external stimulations. HCM, usually inherited in an autosomal dominant pattern with variable expressivity with age‐ and sex‐related, is defined by the presence of left ventricular hypertrophy (LVH) which could not be clarified by abnormal hemodynamics overloading.^1^, ^2^ HCM has a heterogeneous clinical profile, ranging from asymptomatic personnel to symptomatic patients with severe hypertrophic remodelling, inducing diastolic dysfunction and ventricular arrhythmias which are represented as the two dominant features of HCM, leading the main cause of sudden cardiac arrest in young individuals.^3^ As 1980s first discovered the mutation of MYH7 coding the protein of β‐MHC associated with HCM, more than 2000 gene mutations related to 11 kinds of sarcomere proteins have been found to be related to HCM over the past 30 years.^4^ With more and more gene mutations been verified, while around 50% phenotype positive patients still with genotype negative, lead difficult to treat patients directly via the gene mutation of individuals. Therefore, focusing on the downstream mechanisms to find a common target is becoming more and more urgency.

Besides the aetiology of genetic mutation for some specific HCM, the pathophysiological process of cardiac hypertrophy shares a common molecular mechanism of ventricular hypertrophy, which is associated with the sustain cardiac output, and chronically sustained stimulation of undesirable factors would deteriorate the pumping capacity of cardiac, causing multifactorial clinical syndrome, even heart failure. These compensatory and deteriorate changes in cardiomyocyte are associated with re‐expression of foetal genes, which are mainly related to sarcomere contraction, ion signal regulation and activity, and mitochondrial function.^5^, ^6^, ^7^ Increased myofilament Ca^2+^ sensitivity has been identified in animal model and patients with pathological hypertrophy, leading changes of intracellular Ca^2+^ homoeostasis and causing Ca^2+^‐triggered arrhythmias, which could increase force development and adenosine triphosphate (ATP) consumption.^8^, ^9^ Mitochondrial disorganization and dysfunction in cardiac hypertrophy could cause energy supply and demands imbalance.^10^ Clarifying the regulator network of sarcomere contraction, calcium handing and mitochondrial function would benefit the working of finding a new and common target for the treatment of cardiac hypertrophy.

Only 2% of billions DNA bases coding for proteins indicates non‐coding RNAs, including microRNAs (miRNAs) and long non‐coding RNAs (lncRNAs), acting as vital roles in physiology and pathology. MiRNAs, approximately 22 nucleotides in length, are involved in various signalling pathways to regulate pathological cardiac remoulding, such as cardiac hypertrophy, cardiac fibrosis and myocardial infarction.^11^ Meanwhile, lncRNAs could regulate cardiac pathological processes through multiple molecular mechanisms in cis and in trans, which are transcribed RNA molecules >200 nucleotides in length but have no potential for protein coding.^12^, ^13^ More and more studies found that lncRNAs might act an important role to regulate the intracellular calcium handling, metabolic state in various cardiovascular diseases, including HCM (Table 1).^14^, ^15^

**Table 1:** LncRNA candidates in the pathologic progression of hypertrophy cardiomyopathy

LncRNA	Expression	Regulated genes	Pathophysiological mechanism	Methods to generate gain or loss function of lncRNAs	Disease model
Mhrt^21^	**↓**	Brg1	Chromatin remodelling	Mhrt‐ KI	mice‐TAC
Chaer^24^	**↑**	PRC2	Epigenetic checkpoint	Chaer‐KO	mice‐TAC
MEG3^55^	**↑**	miR‐361‐5p/HDAC9	Epigenetic regulation	sh‐MEG3	mice‐TAC/cardiomyocytes‐Ang‐II
DACH1^31^	**↑**	SERCA2a	Ca^2+^ handling	Ad‐Si‐DACH1	mice‐TAC
H19^37^	**↓**	miR‐675‐CaMKIId	Ca^2+^ handling	Ad‐H19	mice‐TAC/cardiomyocytes ‐PE
Plscr4^46^	**↑**	miR‐214‐Mfn2	Mitochondria biological	AAV9‐ Plscr4	mice‐TAC/cardiomyocytes ‐Ang‐II
SNHG1^58^	**↑**	miR‐15a‐5p‐HMGA1	Mitochondria biological	pcDNA‐SNHG1	mice‐TAC/cardiomyocytes ‐PE
TINCR^36^	**↓**	EZH2‐ CaMKII	epigenetic regulation/Ca^2+^ handling	lentivirus‐pcDNA‐TINCR	mice‐TAC
Uc.323^49^	**↓**	EZH2‐CPT1b	Epigenetic regulation/mitochondria biological	lentivirus‐ pcDNA‐ Uc.323	mice‐TAC/cardiomyocytes ‐PE
Ahit^52^	**↑**	SUZ12/PRC2‐MEF2A	Epigenetic regulation/Ca^2+^ handling/mitochondria biological	pcDNA‐Ahit	mice‐TAC/cardiomyocytes ‐PE

In this review, we mainly present an overview to clarify the role of lncRNAs in the pathological process of cardiac hypertrophy further to benefit the work on targeting therapy.

## 2 MOLECULAR BASIS OF CARDIOMYOCYTE HYPERTROPHY ASSOCIATED WITH LNCRNAS

**Figure 1:**
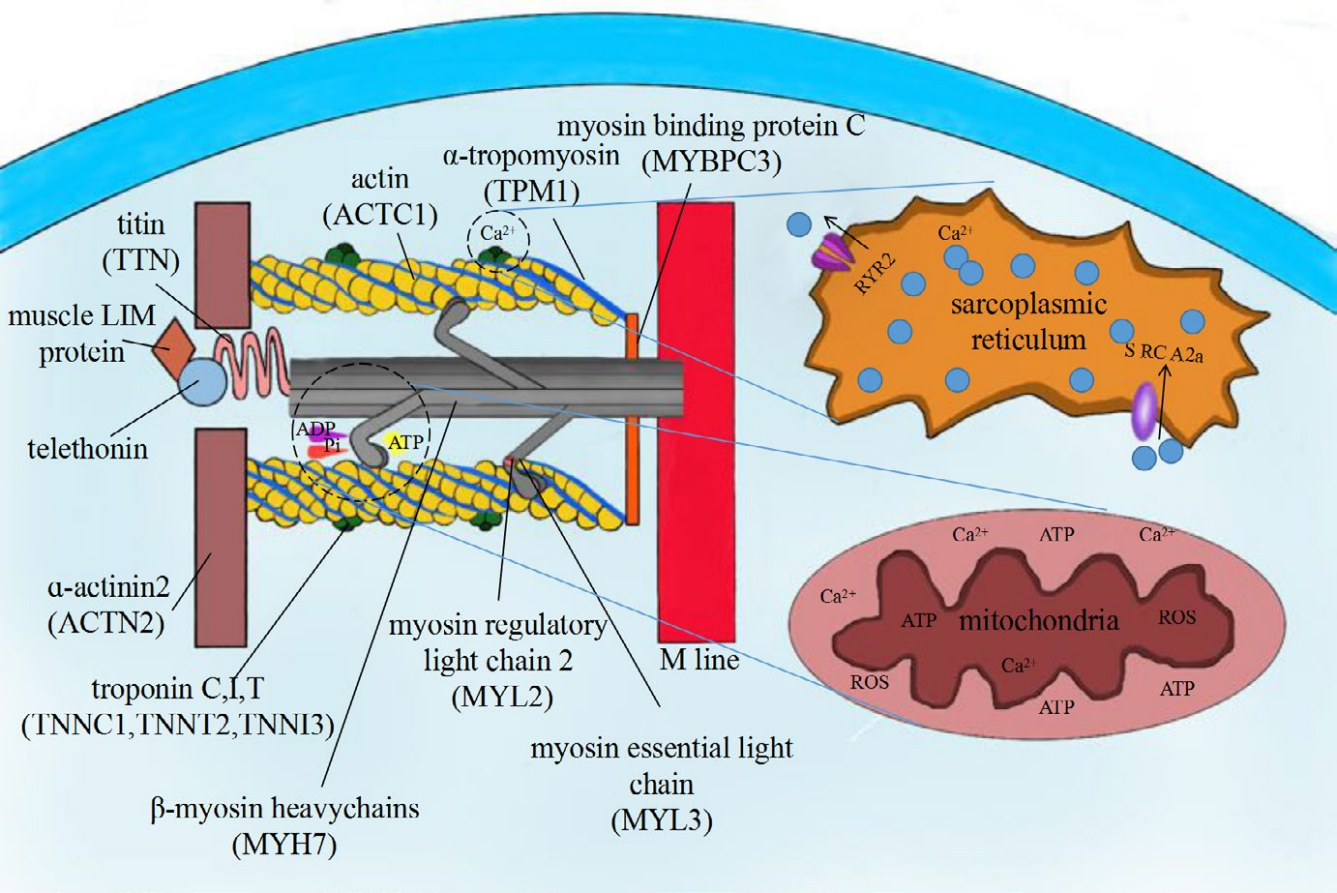
The schematic diagram of sarcomere construction and the relation of which between calcium or mitochondria

Cardiac performance is relied on Ca^2+^ and ATP, to pump blood to meet the energy demands of peripheral tissues, which depends filaments of sarcomere sliding. Sarcomere is the basic functional unit of the cardiomyocyte, made up by the thin and thick filaments containing a set of proteins.^16^ The thin filament is mainly consisting of α‐actin, troponin complex (Tn) containing TnT, TnC, TnI and tropomyosin. The thick filament is mainly composed of myosin heavy chains (MHC), myosin binding protein C (MBPC), and two light chains including regulatory myosin light chain (RLC) and essential light chain (ELC). Myosin filaments are direct link to Z‐line and central of sarcomere, respectively, by protein titin and MBPC (Figure 1).^17^, ^18^

TnC could be activated by calcium‐induced calcium release (CICR), inducing a conformational change in the Tn complex, resulting tropomyosin out of its groove on the actin filament, leading actin filament exposed. Myosin heads also named cross‐bridges, with the binding domain of ATP, can interact with the exposed actin, along with ATP hydrolysing to adenosine diphosphate (ADP) and inorganic phosphate (Pi), which could provide the energy to pull the thin filament towards the centre of the sarcomere. Many sarcomeres contracting in series contract the myofibrils, causing shortening of the whole cardiac fibre (Figure 1).^19^, ^20^

Besides, the Ca^2+^ is critical to generate contraction as myofilament activation and force development is largely dependent on intracellular Ca^2+^.The degree of myofilament Ca^2+^ sensitivity is dynamically regulated by the Ca^2+^ binding affinity of Tn complex.^21^ TnT mutations account for approximately 15% of genotype‐positive HCM cases. This subset of HCM cases are characterized by relatively mild or subclinical hypertrophy with a high incidence of sudden death, which may be associated with increased myofilament Ca^2+^ sensitivity in the mutation.^22^ Which also inspired us that the Ca^2+^ binding affinity of Tn complex may be an integration point to understanding the gene mutation and clinical manifestations.

As mentioned above, the sarcomere contractile cycle is powered by the hydrolysis of ATP relying on the reversible binding of calcium. The HCM has been deemed a ‘disease of the sarcomere’, but the clinical features may be different in the patients even with the same genetic mutation. The growing evidence indicating the LncRNAs participates in the regulation of the complicate network in pathological process of hypertrophy involved the contractile protein expressions, calcium handling and mitochondrial function, which are closely related to the function of sarcomere.

## 3 LNCRNAS IN REGULATION OF PATHOPHYSIOLOGICAL PROCESS OF CARDIAC HYPERTROPHY

### 3.1 LncRNAs reregulate components formation of sarcomere

Nearly, 80% of proteins located in the contractile cardiac myocyte are organized into sarcomeres. While pioneering studies have demonstrated that pathogenic variants of genes encoding sarcomere protein existed among 40%–60% of HCM patients, including β‐MHC (MYH7), MYBPC (MYBPC3), troponin T (TNNT2), troponin I (TNNI3), α‐tropomyosin (TPM1), myosin regulatory light chain 2 (MYL2), myosin essential light chain (MYL3) and actin (ACTC1).^1^, ^2^ So that the malformation of sarcomere is crucial to pathogenesis of HCM, indicating that particular lncRNAs which have been involved in sarcomere protein expression are considering to participant in the regulation of cardiomyocyte hypertrophy.

**Figure 2:**
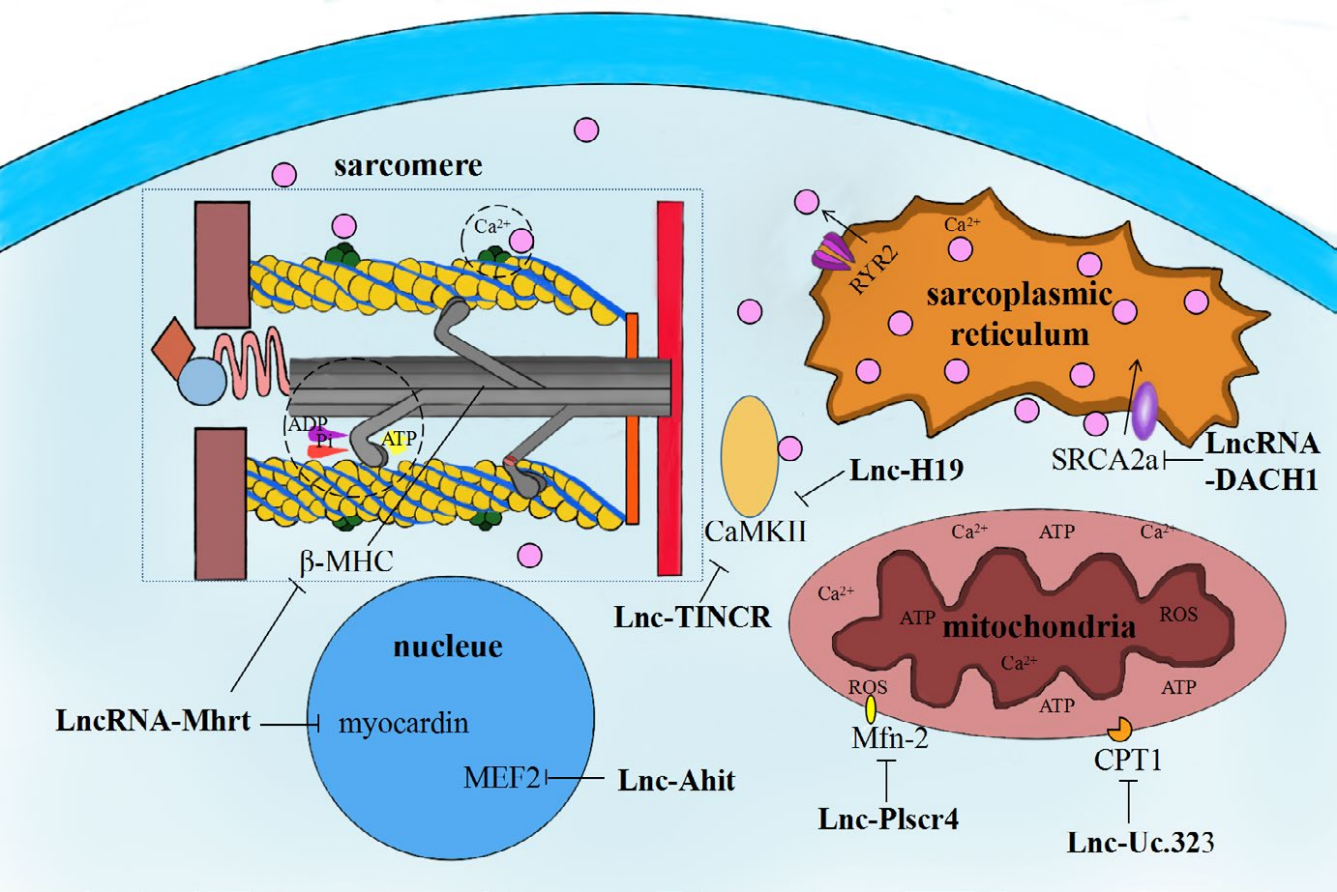
The schematic diagram of the lncRNA and it is targeted protein in cardiomyocytes

LncRNAs, being located in both cytoplasm and nucleus, act as a vital role in HCM, which have been proved the capability to regulate the expressions of sarcomere formatting genes in nuclear, by directly regulation ways. Such as Lnc‐Mhrt, encoded by the gene of *Myh7*, is an inhibitor of pathological hypertrophy, via accessing chromatin remodelling through antagonizes the function of *SMARCA4*.^23^ Besides, Lnc‐Mhrt also could inhibit the expression of myocardin, via targeting miR‐145a‐5p to finally inhibiting the phosphorylation of KLF4, or altering the intracellular location of HDAC5, working as a negative regulator to the pathogenesis of cardiac hypertrophy (Figure 2).^24^, ^25^ So that, once Lnc‐Mhrt is down‐regulated, it is some kind of lossing the inhibition function of *Myh7* expression leading hypertrophic phenotype.

While, lnc‐Chaer interferes with polycomb repressive complex 2 (PRC2) to inhibit histone H3 lysine 27 methylation (H3K27) at the promoter regions of genes involved in stress‐induced pathological gene activation to attenuates cardiac hypertrophy.^26^ Overexpression of lnc‐Chaer would reduce the binding of PRC2 to *Myh7* and *Acta1* genes, resulting in the elevation of such hypertrophic genes, leading to hypertrophy. The lnc‐Chear KO model demonstrated the capability to resist over‐pressure loading induced cardiac hypertrophy.

Therefore, the lncRNAs contributes to maintain the normal expression of hypertrophic genes which are coding foetal contractile isoforms. When cardiomyocytes under pathological stimulations, the level of specific lncRNAs might altering, leading to the re‐expression of *MYH7* and other related genes causing hypertrophic phenotype.

### 3.2 LncRNAs regulate the homoeostasis of mitochondria

Mitochondrial is one of the most important organelles in cardiomyocytes. Nearly, all the biological activity is relied on the homoeostasis and normal function of mitochondria. Mitochondria is directly correlated between cardiac workload and oxygen consumption, which occupy ~30% of cardiomyocyte and generate more than 95% of ATP consumed by the heart.^27^, ^28^ Cardiac contraction is powered by ATP administration, which is produced within mitochondria through oxidative phosphorylation process on fatty acid, while glucose oxidation or glycolysis could also supply some energy under hypoxia condition.^27^ Mitochondria is the multifunctional cell organelle involved in essential functions of cardiomyocytes, including maintain morphology of cardiomyocyte via mitochondrial dynamics process, reactive oxygen species (ROS) accumulation, calcium transportation by uniporter located on mitochondrial membrane, apart from energy generation.^29^ Pathological hypertrophy is associated with mitochondria dysfunction.^27^ The emerging evidence suggested that lncRNAs contribute to the synchronization of cellular biological pathways in HCM, via regulating the homoeostasis of mitochondria.

A key research from Song et al^30^ demonstrated the role of mitochondrial dynamics process in inducing cardiomyopathy and proved that the impairment of mitochondrial dynamics would lead to cardiac hypertrophy and heart failure. Besides, the mitofusin 1 (Mfn1) and mitofusin 2 (Mfn2) double knock out induced more mitochondrial fission, resulting in eccentric hypertrophic modelling. Besides, another research from Papanicolaou et al demonstrated the absence of Mfn2 in cardiomyocyte is associated with delayed mitochondrial permeability transition and local generation of ROS, resulting in cardiac hypertrophy.^31^, ^32^ So that, the homoeostasis of mitochondrial dynamics process is essential to maintain cardiomyocyte normal morphology. Lv et al found that the lnc‐Plscr4 participant in the regulation of miR‐214‐Mfn2 axis to main mitochondrial dynamics process. Their findings revealed the inhibition of lnc‐Plscr4 inducing hypertrophy, while overexpression attenuate the hypertrophy both from angiotensin II treated cardiomyocytes and transverse aortic constriction treated mice.^33^ Moreover, Anusha et al^32^ reviewed that miRNAs were involved in the regulation of mitochondrial dynamics. So that, a series of non‐coding RNAs, such as lncRNAs and miRNAs, have been identified to be involved in the mitochondrial dynamics controls, which may be a potential therapeutic to intervene the cardiac diseases. However, according to common sense, the interactions between enrolled lncRNAs and miRNAs need to be further demonstrated.

Beyond the morphology relationship between mitochondria and cardiomocyte, the metabolic alternations are another character during cardiac hypertrophy. Metabolic remodelling in pathological hypertrophy with shift from predominant long‐chain fatty acid utilization to glucose utilization will result in an shortage in ATP consumption.^34^ Carnitine palmitoyl transferase I (CPT1) need to be recruited in the first step in the transportation of fatty acids into mitochondria, which is encoded by *CPT1b* gene in heart and muscle cells. The activity of *CPT1b* is considered to guiding fatty acid oxidation.^35^ While, lnc‐Uc.323 has been documented it could regulate the expression of *CPT1b* via interacting with its coactivator of zeste homolog 2 (EZH2), which bound to the promoter of *CPT1b* via H3K27me3 to induce *CPT1b* down‐regulation, protecting cardiomyocytes against hypertrophy (Figure 2).^36^ The lnc‐Uc.233 depletion mice presented a severe hypertrophic heart, providing evidence on the association between lncRNAs and metabolic disorder in regulation of cardiac hypertrophy.

### 3.3 LncRNAs controls the function of calcium channels

Arrhythmia is always combined with HCM, which may be associated with the Ca^2+^ mishandling. Several studies have identified some gene mutations associated with Ca^2+^ regulatory which contributed to cardiac hypertrophy, including phospholamban (PLN), ryanodine receptor 2 (RYR2) and junctophilin 2 (JPH2).^37^ Calcineurin overexpression mice model is a typical standard model to induce HCM. While, it is a strong evidence to emphasize that Ca^2+^ mishandling is crucial to generate cardiomyocyte hypertrophy. So that the electrical remodelling is also a great part of biological processes changing among HCM.^22^, ^37^, ^38^ Here, we intend to identify the roles of LncRNAs in Ca^2+^ handling network, which may contribute to hypertrophic phenotype by sustained Ca^2+^ mishandling induced electrical activity.

During the cardiac action potential, Ca^2+^ enters the cell and triggers Ca^2+^ release from the sarcoplasmic reticulum (SR). The raising concentration of Ca^2+^ in the cytoplasm allowing Ca^2+^ to bind to the TnC, which then switches on the contractile machinery. Ca^2+^ transport out of the cytosol for relaxation occurring by four pathways, involving SR Ca^2+^‐ATPase, sarcolemmal Na+/Ca^2+^ exchange, sarcolemmal Ca^2+^‐ATPase or mitochondrial Ca^2+^ uniporter.^21^, ^39^ So that, the dysfunction of four types of Ca^2+^ channels mentioned above would lead to the pathophysiological process of hypertrophy.

SR ATP‐driven Ca^2+^ pump (SERCA2a) is an enzyme located on the SR membrane that pumps calcium into the SR, and the function of SERCA2a is closely related to calcium transient amplitude and calcium store capacity in cardiac myocytes.^40^ Lnc–Dach1 (dachshund homolog 1) played as negative role in regulating calcium handling, which binds to SERCA2a and enhances its ubiquitination leading to the reduction of SERCA2a protein and impairing calcium handling and cardiac function (Figure 2).^41^ The lnc‐Dach1 increased in hypertrophic cardiomyocyte and impaired the SERCA2a induced calcium release by RyR2, resulting in weaker contraction and pathological hypertrophic remodelling.

Ca^2+^/calmodulin‐dependent protein kinase II (CaMKII) is associated with phosphorylation of a number of Ca^2+^ handling proteins with α, β, γ, δ isoforms, relating to the activation of the SERCA2a and RyR2, the disturbing of which would lead inappropriate membrane potential depolarizations, and the higher activity of CaMKII would cause cardiomyocyte hypertrophy.^42^, ^43^, ^44^, ^45^ Lnc‐TINCR could regulation the expression of CaMKII via epigenetically silencing, as knockdown of lnc‐TINCR was found to reduce EZH2 occupancy and H3K27me3 binding in the promoter of CaMKII in cardiomyocytes (Figure 2).^46^ Lnc‐H19 as the precursor of miR‐675, which inhibit the expression of CaMKIIδ, is a negative regulator of cardiomyocyte hypertrophy (Figure 2).^47^ Calcineurin is serine/threonine protein phosphatase regulated by CaMKII, which plays an important role in the regulation of pathological hypertrophy.^48^ Calcineurin could reciprocal repress with miR‐133 to regulate cardiac hypertrophy.^49^ Jiang et al^50^ found that lncRNA‐ROR mediated pathological hypertrophy via decreasing the expression of miR‐133, which could influence the handing of calcium.

MEF2 (myocyte enhancer‐binding factor 2) family, including MEF2A, MEF2B, MEF2C and MEF2D, have the ability to bind A/T‐rich sequences within gene promoter of muscle creatine kinase, which is a critical regulator of cardiac development and cardiac gene expression. MEF2 could mediate Ca^2+^/calmodulin‐dependent signalling to regulate Ca^2+^ signalling pathway, and regulate mitochondrial genes transcription to involve the bio‐function of mitochondrial.^51^, ^52^ LncRNA‐Ahit is a neighbouring gene of MEF2A, which regulate chromatin remodelling via direct binding with SUZ12 (suppressor of zeste 12) and recruiting PRC2 to promote of H3K27me3 on the MEF2A promoter region, resulting against cardiac hypertrophy. Although reduction of MEF2A was the most obvious after down‐regulated Ahit, all MEF2 members were induced, including MEF2D.^53^ MEF2D could regulate the gene NADH dehydrogenase 6 (ND6) expression, which directly modulate complex I activity of mitochondrial, further affecting a number of key mitochondrial functions (Figure 2).^54^ HDACs act a link role between CaMK and MEF2, in other words, which could act as a media role between calcium handing and mitochondria bio‐function.^55^ LncRNA‐MEG3 activated by STAT3 is a positive regulator in pathological hypertrophy through miR‐362‐5p/HDAC9.^56^ Thus, LncRNA‐Ahit and LncRNA‐MEG3 could regulate cardiac hypertrophy involved in the mitochondria bio‐function and calcium signal pathway via regulating the integration protein of MEF2.

## 4 LNCRNA: A POTENTIAL TARGET FOR CARDIAC HYPERTROPHY

The current pharmacologic therapy remains alleviating the symptoms of HCM patients rather than focusing on the underlying cause, such as using β‐adrenergic receptor blocker to blunt the heart rate and increase diastolic filling time. With more and more genetic analyses and mechanistic studies positing the sarcomere contraction as a central mediator of cardiac hypertrophy, specific therapies which could regulate the function of sarcomere have been raised up, one of which, named MYK‐461 (mavacamten), could selectively reduce myosin ATPase activity and are currently in human clinical trials.^57^

With much progress been made in elucidating the underlying mechanisms of pathological hypertrophy, many new insights on targeted therapies for cardiac hypertrophy have been proposed. LncRNAs, with the advantages of expressed with tissue and disease specificity, stable in body fluids, and smaller than the coding protein, are more attractable as diagnosis biomarkers and therapeutic targets for cardiac hypertrophy. Silenced Lnc‐MEG3 or overexpression Lnc‐Plscr4 and Lnc‐CHAR could attenuate the increasing size of cardiomyocytes induced by angiotensin II (Ang‐II).^33^, ^56^, ^58^ Overexpression of Lnc‐H19, Lnc‐SNHG1, Lnc‐uc.323 or loss‐expression of Lnc‐Ahit and Lnc‐Chast could reduce the cardiomyocytes size in response to phenylephrine.^36^, ^47^, ^59^, ^60^ Conditional knockout of Lnc‐DACH or overexpression Lnc‐TINCR resulted in increasing calcium transient, and improving cardiac function after transverse aortic constriction in mice.^41^ Inhibition of Lnc‐Chaer expression before the onset of pressure overload substantially attenuates cardiac hypertrophy on mouse model.^26^

## 5 VALIDATION OF A LNCRNA‐BASED GENE THERAPY ON NON‐VIRAL AND VIRAL CONCEPTIONS

Several approaches have been applied to generate the silence and overexpression oligonucleotide for lncRNAs. Simply, the lncRNA siRNA has been used to inhibit the site targeting for lncRNA. Besides, the anti‐sense oligonucleotide (ASO) has been widely introduced to impair the function of lncRNAs. Beyond cardiovascular system, the application of ASO for lncRNA has been confirmed a promising therapeutic method for osteoarthritis, hepatology disease and cancer. While the lncRNA minics has been used to expression specific oligonucleotide (Table 1). Moreover, some gene‐editing method has also been taken into knock out particular lncRNA DNA sequences, such as CRISPR/Cas9 techniques. Based on the oligonucleotide design protocols, researchers were able to demonstrate the capability of lncRNA targeting therapy for cardiac hypertrophy in vivo on small animal models.

These studies have proved the efficient on therapy pathological hypertrophy via gain or loss function of the lncRNAs in vitro or in vivo cardiac hypertrophy model, which supply a new perspective for the treatment of cardiac hypertrophy. Now, a series of microRNAs‐based therapies have been applied for clinical trails. However, the lacking of big animal experiments limited the expanded application for lncRNA therapy. Only H19 lncRNA has been tested in pigs to prove the expression of lncRNA H19 increased in two abdominal aortic aneurysm mouse models by a low‐density lipoprotein receptor knockout pig model.^61^ So that more experiments designed for large animal are essential before launching clinical trails.

**Figure 3:**
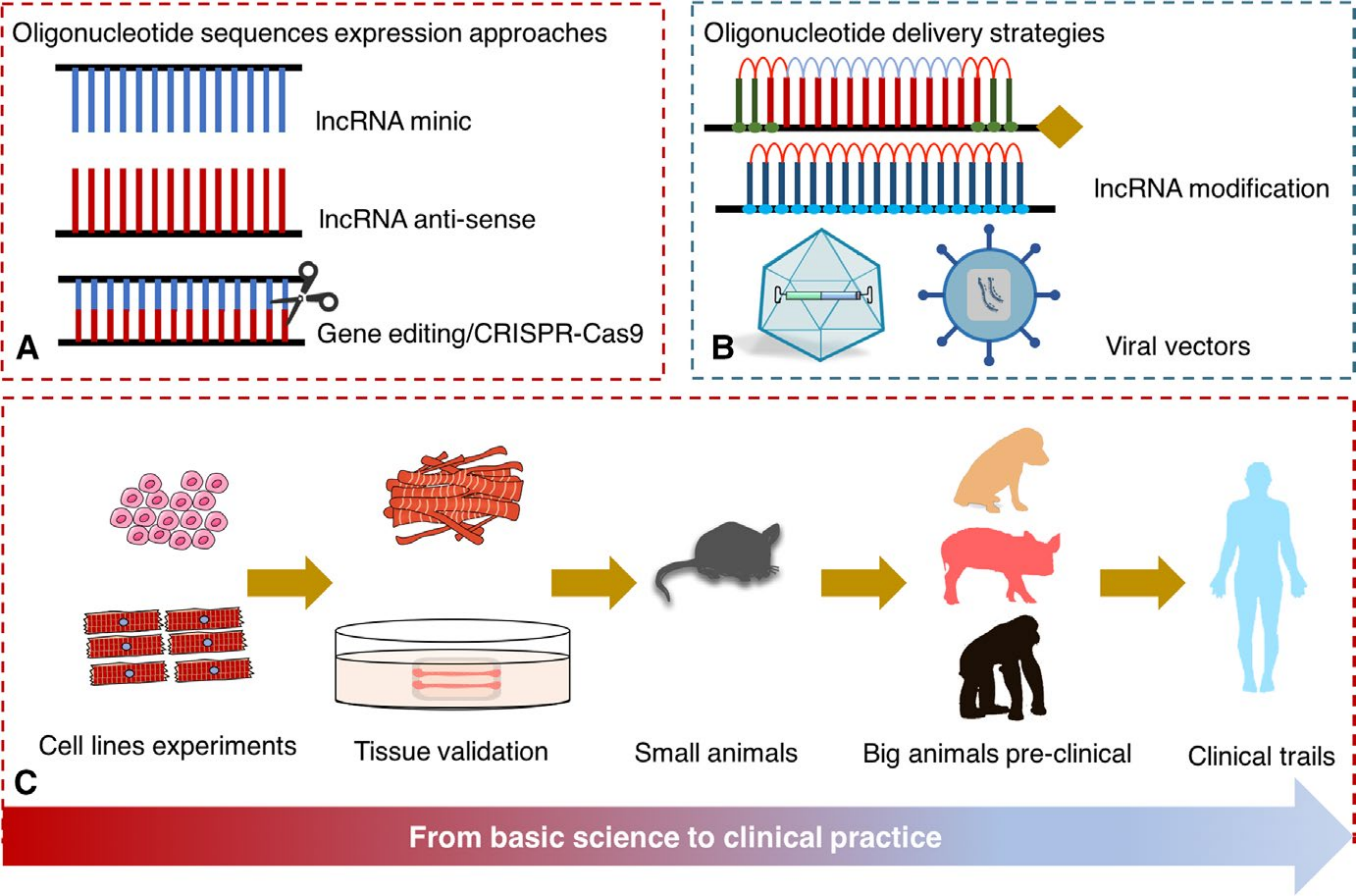
The common protocol to validate the lncRNA‐based gene therapy to clinical applications. A, The design strategies for the purpose of targeting lncRNAs. B, The non‐viral and viral methods to establish a delivery system for lncRNA‐based gene therapy. C, The validation program of lncRNA‐based gene therapy from cell lines and cardiomyocytes’ tissue verification, small and big animals’ validation and finally launching a clinical trail

However, the simply designed oligonucleotide is not suitable for large mammals’ tissue‐specific expression. According to the evidence on the confirmation of therapeutic efficacy of lncRNAs on cardiac hypertrophy, newly oligonucleotide delivery system is required to be developed to silence or overexpress a particular lncRNA in cardiovascular system. Typically, there are two novel methods to delivery designed oligonucleotide as viral and non‐viral concepts.^62^ For the viral concept, several vectors including adenovirus, adeno‐associated virus (AAV) and lentivirus (Table 1). While the non‐viral concept involved liposomes or nanoparticles approaches to delivery oligonucleotide to heart tissue. Besides, chemical modification, including basic cholesterol modification, 2′‐O‐(metoxyethyl) (2′MOE), 2′fluoro (2′F), locked nucleic acid and antagomirs strategy, has been widely used for in vivo lncRNA delivery.^63^ Beyond lncRNA, AAV seems to be the most promising delivery strategy for continuous expression oligonucleotide in heart tissue. Two researches from William T. Pu’s group used AAV to delivery AIP peptides and gene *Tafazzin* in mice to rescue the pathological phenotype of catecholaminergic polymorphic ventricular tachycardia and Barth syndrome,^64^, ^65^ which has proved the advantages of applying AAV system in gene therapy within cardiovascular system.^66^ Besides, the AAV vector has been approved by FDA to clinical application for gene therapy at 2017.^67^ At present, more than 30 clinical trails are undergoing on disease treatment of brain, spinal cord, eye, liver and muscle. So that, taking the advantages of various delivery vectors, targeting cardiac hypertrophic lncRNAs would be a potential way to terminate or even reverse pathological hypertrophy remodelling. The Figure 3 demonstrated the common protocol to validate the lncRNA‐based gene therapy to clinical applications (Figure 3).

## 6 CONCLUSION

Over the past several decades, the molecular mechanisms on cardiac hypertrophy have made a further progression. However, there are still some big dilemmas to fully elucidating the pathological mechanisms for better treatment of cardiac hypertrophy. More and more evidence illustrated that lncRNAs, such as Mhrt, Plscr4 and H19, could act as an important participant in the complicated network in regulating the pathological process of hypertrophy, which involves in the functional homoeostasis among sarcomere, mitochondria and intracellular calcium transition, making them as a promising target for treatment of cardiac hypertrophy. This review demonstrated the function of lncRNAs in the processes of pathology hypertrophy, aimed to provide a new insight to explore the pathological mechanisms and to find novel therapeutic targets for disease modulation and prevention in cardiac hypertrophy. Besides, the review presented current strategies to provide gene therapy and the program to validate a lncRNA‐based therapeutic approach from bench site to bedside.

## CONFLICT OF INTEREST

The authors report no conflict of interest. The authors alone are responsible for the content and writing of the paper.

## AUTHOR CONTRIBUTIONS

**Lei Liu:** Conceptualization (supporting); Investigation (lead); Resources (lead); Writing‐original draft (equal). **Donghui Zhang:** Project administration (lead); Supervision (equal); Validation (equal); Writing‐review & editing (equal). **Yifei Li:** Conceptualization (lead); Supervision (equal); Validation (equal); Visualization (equal); Writing‐original draft (equal); Writing‐review & editing (equal).
